# Daily Consumption of Kombucha Influences the Urinary and Plasma Metabolome in a Healthy Human Cohort

**DOI:** 10.1002/fsn3.71020

**Published:** 2025-10-13

**Authors:** Amanda J. Lloyd, Robert J. Nash, Alexander N. W. Taylor, Alina Warren‐Walker, Courtney Davies, M. J. Pilar Martinez Martin, Odin M. Moron‐Garcia, Alison Watson, Laura Lyons, Mark J. Pavey, Thomas Wilson, Manfred Beckmann, Kyriaki Remoundou, Nigel Holt

**Affiliations:** ^1^ Department of Life Sciences Aberystwyth University Aberystwyth UK; ^2^ Phytoquest Limited, IBERs Plas Gogerddan, Aberystwyth UK; ^3^ Department of Psychology Aberystwyth University Aberystwyth UK; ^4^ Conwy Kombucha Limited Conwy UK; ^5^ Aberystwyth Business School Aberystwyth University Aberystwyth UK

**Keywords:** black tea, fermented tea, flow infusion electrospray ionization mass spectrometry, green tea, traditional kombucha

## Abstract

This study investigated a traditionally brewed, organically produced kombucha, which may contain antioxidants, polyphenols, and bioactive compounds resulting from fermentation. While kombucha is marketed as a functional drink, there is limited empirical research on the drink's functional benefits. Additionally, much kombucha sold in the global marketplace is produced using instant mixes, pasteurization, filtration, and top carbonation, creating a shelf‐stable ambient product at significantly lower cost than fresh, refrigerated traditional Kombucha. An 8‐week randomized, double‐blinded, parallel trial investigated the effects of daily kombucha (330 mL) consumption on urinary and plasma metabolomics in healthy adults aged 18–71. Participants of mixed gender and ethnicity consumed either a trial canned kombucha or a placebo (flavored water). First Morning Void (FMV) urine and fasting venous blood samples were collected before and after the trial period. Urine samples were analyzed using Flow Infusion Electrospray Ionization Mass Spectrometry (FIE‐MS), while plasma short‐chain fatty acids (SCFAs) were quantified using Gas Chromatography–Flame Ionization Detection (GC‐FID). The urinary metabolomic profile showed an increase in metabolites linked to kombucha‐derived polyphenols and microbial fermentation, such as dihydroferulic acid and arabitol. Plasma analysis revealed a significant reduction in acetic acid and a marginal decrease in isoButyric acid after kombucha consumption. These findings highlight the complex interplay between fermented food consumption and human metabolism.

## Graphical abstract

An 8‐week randomized, double‐blind trial tested daily canned kombucha intake (330 mL) in healthy adults. Urinary metabolomics revealed increased polyphenol‐ and fermentation‐related metabolites, while plasma analysis showed reduced acetic acid and slightly lower isoButyric acid. Findings suggest kombucha consumption alters human metabolism, though health effects remain unclear.

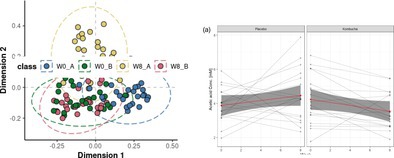

## 1 Introduction

Tea is made from the leaves of the plant *Camellia sinensis L*. and is a widely consumed UK beverage, known for its health‐promoting properties (Pan et al. 2022). The regular consumption of tea promotes wellness, in particular, improved aging and a reduction of cardiovascular diseases, cancers, hepatopathy, obesity, and diabetes mellitus (Samanta 2022; Cabrera et al. 2006). Furthermore, epidemiological studies have linked tea consumption to lower levels of emotional distress (Lange et al. 2022).

Among tea‐based products, kombucha—a fermented beverage traditionally made from green or black tea using a symbiotic culture of bacteria and yeast (SCOBY)—has garnered significant attention for its potential health and wellness benefits (Massoud et al. 2024). Originating from ancient Eastern traditions, kombucha is globally marketed as a functional beverage. Kombucha consumption has increased substantially in recent years due to growing consumer interest in food and beverages with functional properties (Kim and Adhikari 2020). Despite marketing claims, the microbial composition of kombucha varies widely depending on production methods, pasteurization, storage, and ingredient choice (Kitwetcharoen et al. 2023; Huang 2024). Not all kombuchas are probiotic, as this status requires demonstrated health benefit, specific microbial strains, and evidence of viability at the point of consumption. The microbial composition and health effects may vary across kombucha products depending on ingredients and processing methods. A recent study among UK consumers revealed high health consciousness and willingness to try functional foods and drinks such as kombucha (Remoundou et al. 2025). Despite its popularity, many of kombucha's purported health benefits remain anecdotal and require scientific validation.

Kombucha's fermentation process may enhance its nutritional profile by introducing potentially beneficial microbial metabolites; however, probiotic content is not guaranteed and must be specifically validated. Kombucha's fermentation can also result in an enrichment of antioxidants and anti‐inflammatory compounds, which, after consumption, may reduce oxidative stress and inflammation (Soares et al. 2023; Prajapati et al. 2024). Additionally, studies suggest kombucha consumption may lower cholesterol levels and blood pressure, contributing to improved metabolic health (Massoud et al. 2024). Yet, bridging the gap between traditional claims and empirical evidence remains essential, and innovative analytical approaches are needed to fully elucidate kombucha's effects (Huang 2024).

Recent randomized controlled trials have provided some evidence for kombucha's health effects. A recent randomized controlled trial investigated the effects of kombucha (a green and black tea mix) and placebo on individuals with excess body weight, focusing on intestinal health, gut microbiota composition, and serum metabolomics (Fraiz et al. 2024). Both groups showed improvements in gastrointestinal symptoms, with the kombucha group reporting fewer issues related to motility. No significant differences in microbiota composition were observed between groups. However, only the control group exhibited increased fecal pH, lactulose/mannitol ratio, and zonulin levels, indicating potential worsening of intestinal permeability. Regarding serum metabolomics, the kombucha group displayed unique metabolites, such as diethyl malonate, suggesting positive influences on the serum metabolome. Additionally, another study explored the impact of black tea kombucha consumption on gut microbiota of healthy humans over 8 weeks (de Campos Costa et al. 2024). The study found that kombucha consumption modulated the gut microbiota composition, with more pronounced benefits observed in the obese group. More recently, a controlled clinical trial investigating the effects of a four‐week kombucha intervention in healthy adults consuming a Western diet showed subtle but measurable shifts in gut microbiota composition, including enrichment of *Weizmannia coagulans*, a probiotic species associated with short‐chain fatty acid (SCFA) production after kombucha consumption (Ecklu‐Mensah et al. 2024). These findings suggest that regular intake of kombucha may influence gut microbiota composition and metabolic outputs, particularly in individuals with obesity, potentially leading to improved metabolic health, although inter‐individual variability and study duration remain important considerations.

Urinary metabolomics offers a non‐invasive, high‐throughput approach to monitoring biochemical shifts, capturing both endogenous metabolism and dietary influences. The technique is particularly suited to studying the impact of dietary interventions like kombucha consumption. The quantification of urinary metabolites offers a non‐invasive method to monitor chemical dynamics in clinical and research settings. Flow Infusion Electrospray Ionization Mass Spectrometry (FIE‐MS) has emerged as a high‐throughput, non‐targeted metabolite fingerprinting technique suitable for this purpose, enabling efficient exploration of complex urinary fingerprints (Draper et al. 2013; Lloyd et al. 2011; Beckmann et al. 2008). When combined with multivariate modeling, this approach provides valuable insights into dietary and metabolic interactions. Additionally, SCFA analysis of plasma complements urinary metabolomics by offering targeted insights into gut microbial activity and host–microbiota metabolic interplay. As key microbial fermentation by‐products, SCFAs serve as functional biomarkers of dietary fiber fermentation and microbial balance. Their quantification enables evaluation of systemic exposure to gut‐derived metabolites, helping to elucidate the broader metabolic effects of interventions such as kombucha consumption.

This study aimed to address the gap between anecdotal claims and evidence‐based outcomes by evaluating the effects of daily traditional kombucha consumption on urinary metabolomics and SCFAs in plasma in a healthy cohort. Using a randomized controlled design, we hypothesized that kombucha consumption would result in significant changes in urinary metabolite fingerprints and SCFAs in plasma, reflecting enhanced metabolic and microbial activity compared to a placebo. By integrating advanced metabolomics with machine learning and statistical approaches, this study sought to uncover kombucha's multifaceted biochemical effects, providing robust evidence for its health‐promoting properties and advancing our understanding of functional beverages as a dietary intervention.

## 2 Material and Methods

### 2.1 Participants

This was a double‐blind, randomized, placebo controlled parallel human clinical trial (registered in the ISRCTN registry (https://www.isrctn.com/) under registry number ISRCTN70525386) approved by the Research Ethics Panel at Aberystwyth University. Participants were recruited by the Well‐being and Health Assessment Research Unit (WARU) at Aberystwyth University and the study was carried out in accordance with the Declaration of Helsinki, where participants gave written informed consent. The pre‐screening for the first volunteers started in March 2024 and the study was completed in July 2024. The trial was conducted at WARU and the participants followed the intervention trial at home and visited at baseline and after the trial (8 weeks) for measurements, tasks, venous blood draws, urine collections, and self‐report questionnaires.

Recruitment was carried out by the WARU research technicians largely via social media (Facebook), with advertisements posted in community groups local to Aberystwyth (within a 40‐min commuting distance to the University). Recruitment was also conducted in person, and Participant information sheets were also circulated via email to different University departments. Participant information sheets were also shared among a pre‐existing database of people who had shown interest in WARU's previous research activities. Participants were asked to e‐mail WARU to voice initial interest, with this being followed up with a pre‐screening phone call to check eligibility prior to enrolling the individual in the study. Participants were then invited to an in‐person induction, where their height, weight, waist, and hip circumference were obtained, their consent forms were signed, and they were talked through and provided with their urine home sampling kits.

Participants were recruited according to eligibility criteria. Inclusion criteria included: consenting participants over 18 years of age and under 80 years of age; participants who are able to commit to multiple visits to WARU; subjects who can provide venous blood samples, urine samples, and saliva samples; participants able to provide written informed consent prior to performing any study procedures. Exclusion criteria included: participants with a diagnosis of Alzheimer's disease or other forms of dementia; participants taking medication for the treatment of dementia (such as acetylcholinesterase inhibitors (Aricept, Excelon), memantine (Namenda) or other medications with similar mechanisms of action) or medical foods (such as Cerefolin, Souvenaid, Axona) for the treatment of dementia; participants who are pregnant or lactating; participants with a medical condition or disease that is life‐threatening; participants diagnosed with diabetes; participants already consuming pro‐biotics who will not comply with the 4‐week washout; participants with any cardiovascular diseases; participants with severe physical illnesses (e.g., fibromyalgia); participants experiencing hypertension (high blood pressure); participants with endocrine disorders; participants suffering from substance abuse; participants who heavily smoke (> 10 cigarettes/day); and any medication known to affect the HPA hypothalamic‐pituitary‐adrenal axis. If eligible, participants were allocated to two groups using computer‐generated random number tables: Kombucha or placebo. The study was double‐blinded, so researchers and intervention participants were unaware of the allocation until intervention and analysis completion.

### 2.2 Sample Size Calculation

The findings reported in this paper are part of a larger trial where self‐reported information was collected, including the Psychological Well‐Being Scale (PWB), EuroQol‐5D‐5L, Depression Anxiety Stress Scale (DASS‐21), Positive and Negative Affect Scale (PANAS)‐GEN, and the Visual Analogue Scale (Lovibond and Lovibond 1995; Watson and Clark 1999; Keyes 1995; EuroQol‐Group 1990). Additionally, the Maastricht Acute Stress Test (MAST) was conducted together with cortisol measurements in saliva (Shilton et al. 2017; Smeets et al. 2012). The analysis of this data suggested multiple minimum differences (small, medium, and large) between the intervention and placebo arms that were considered clinically meaningful for the primary and secondary outcomes (e.g., self‐report, MAST, metabolomics, etc.) due to the experimental mixed model design. Furthermore, high‐throughput metabolomics is about hypothesis generation, rather than looking for clinically meaningful outcomes. For a large effect size (*d* = 0.8), each group (kombucha and placebo) would require approximately 26 participants to achieve a significance level (*α* = 0.05) and 80% power. This resulted in a total sample size of approximately 51 participants required for the trial.

### 2.3 Study Design: Dose and Type

The study was split into two experimental sessions (0 and 8 weeks) where the participants were randomized to one of two arms of kombucha (330 mL can) or placebo (can of cherry flavored sparkling water, near‐matched by taste and appearance of a commercial kombucha) daily supplementation for 8 weeks. The Kombucha and flavored water (ingredients in Appendix S1) were consumed throughout the day as the participants desired. To evaluate compliance, participants were requested to note any missed cans and to bring back the remaining cans. The prototype kombucha provided by Conwy Kombucha Ltd. was brewed using organic green tea and black tea, with cane sugar as the carbon source. The fermentation lasted 10 days at ambient temperature using a symbiotic culture of bacteria and yeast (SCOBY). The Kombucha used was naturally carbonated during primary fermentation, and secondary fermentation was inhibited by refrigeration and top CO_2_ pressure. This approach was adopted to maintain batch stability over the 8‐week intervention period. The final intervention beverage was non‐pasteurized.

### 2.4 Anthropometric Measures

Body weight (using SECA 799 Electronic Column Scales to the nearest 0.1 kg) and height were measured (using a Holtain Stadiometer to the nearest cm) to allow the body mass index (BMI) to be calculated and compared to ranges set out by the WHO (World Health Organization 2006). Waist circumference (midway between the lower rib and the iliac crest on the midaxillary line) and hip (level of the widest circumference over the great trochanters) were taken to the nearest 0.1 cm, using an ergonomic circumference measuring tape over bare skin, whenever possible. Triplicate measurements were made, and the mean was calculated, allowing the waist‐to‐hip ratio to be calculated and compared to published ranges (Hollmann et al. 1997; Park et al. 2015). All measurements were done in the morning by the researcher after the participants had been fasting for at least 8 h.

Blood pressure was measured using an Omron HEM‐907 calibrated automated sphygmomanometer, with the participant seated and relaxed for at least 5 min in a quiet environment. Measurements were taken on the participant's dominant arm, supported at heart level, using an appropriately sized cuff to ensure accuracy. Triplicate readings were recorded at 1‐min intervals, and the mean systolic and diastolic blood pressure values were calculated. Measurements were performed at the start of the appointment to minimize the potential effects of the clinical visit or other activities on blood pressure readings, following guidelines set out by the European Society of Hypertension (Mancia et al. 2007). If a reading of 140/90 mmHg and 160/100 mmHg was observed by a researcher (on the test day), then the participants were unfortunately not eligible to participate. These numbers were taken from the British Heart Foundation (British Heart Foundation 2023). This was because we anticipated participants experiencing short‐term elevations in blood pressure during the hand‐immersion trials of the MAST test (not reported in this manuscript).

### 2.5 Blood Collections

Participants came to WARU fasted between 07:00 and 10:00 am, having been told to drink plenty of water prior to their visit and to remain hydrated. A > 8 h fasted blood sample was taken intravenously following standardized procedures. Bloods were collected in a 4 mL SST serum tube and a lithium‐heparin plasma tube. After the tubes were filled with blood, they were inverted 5–10 times to activate the clotting and left to stand for 20–30 min (SST Only). They were then centrifuged at 2000 rpm, 4°C, for 10 min. The supernatants were aliquoted into new 2 mL labeled eppendorfs. They were then stored at −80°C until analysis.

### 2.6 Collection of Urine Samples

Each participant collected first morning void (FMV) urine samples at home using the vacuum transfer system as described in Lloyd et al. (2020). Written instructions on how to collect FMV urine samples were provided. All samples were stored at home at 4°C and then brought to WARU. Duplicate tubes were centrifuged at 4°C and 4000 rpm for 5 min, rendering the samples acellular. The supernatants from the two tubes were aliquoted into clean and labeled 4 mL cryotubes and then stored at—80°C for analysis.

### 2.7 Processing of Urine Samples

Urine samples were prepared and adjusted as reported previously (Beckmann et al. 2020; Lloyd, et al. 2019a; Lloyd, et al. 2019b; Lloyd et al. 2020). In brief, all urine samples were normalized by Refractive Index (RI) prior to analysis to account for differences in fluid intake by participants and to ensure that all mass spectrometry (MS) measurements were made within a similar dynamic range within the linear range of the instrument. Samples were defrosted overnight at 4°C, centrifuged (1600 ***g*** for 5 min at 4°C), placed on ice and aliquots of thawed urine (1000 μL) were transferred into labeled 2 mL eppendorf tubes. The remaining sample were returned to a −20°C freezer. An OPTI handheld refractometer (Bellingham Stanley Brix 54 Model) was used to record the specific gravity (SG). Using these data, aliquots of the required amounts of urine from centrifuged 2 mL eppendorf tubes and ultra‐pure (18.2 Ω) H_2_O were transferred into new tubes for extraction; this ensured that all samples had the same RI. Adjusted urine samples were extracted with an equal volume of100% methanol.

### 2.8 Flow Infusion Electrospray Ionization Mass Spectrometry (FIE‐MS) Analysis of Urine

FIE‐MS analysis was conducted with an Exploris 120 mass analyzer coupled with a Dionex Vanquish ultra high‐performance liquid chromatography (UHPLC) system (Thermo‐Scientific), measuring ion intensities within the m/z range of 55–1200. The mobile phase was methanol:water (70:30). Metabolite fingerprints were generated in both positive and negative ionization modes, in a single run. The data obtained was subjected to spectral binning followed by multivariate analysis (Lloyd et al. 2025).

All samples were randomized to minimize batch effects. Samples (20 μL) were injected into a flow of 100 μL min^−1^ methanol:water (70:30, v/v). Ion intensities were acquired between m/z 55 and 1200 for 3.5 min at a resolution setting of 120,000, resulting in 3 (±1) ppm mass accuracy. Tuning and ESI source parameters were set according to the manufacturer's recommendations. Following data acquisition, Chromeleon.cmbx files were first exported to .raw files and then converted to the .mzML open file format and centroided (Martens et al. 2011) using msconvert (TransProteomicPipeline) (Chambers et al. 2012). Spectral binning was applied using the R package binneR (Finch et al. 2022), and then standard post‐acquisition processing routines were applied, including occupancy and QC filtering. Putative molecular formulas were generated by using MZedDB (Draper et al. 2009), an Aberystwyth University database for accurate mass annotation.

Intra‐batch variance was removed, adjusting intensity values by the mean value of the respective analytical block (where each block contains an equal representation of the total biological variance). Prior to statistical analysis, data were normalized to the total ion count (TIC) of the sample. For multivariate analysis, all samples and features were used.

Supervised Random Forest (RF) classification was implemented using the randomForest package in R (R Core Team 2013). For all RF models, the number of trees (*ntree*) used was 1000 and the number of variables considered at each internal node (mtry) was the square root of the total number of variables. RF classification models were plotted following multi‐dimensional scaling (MDS). Proximity measures for each individual observation were extracted from RF models and scaled coordinates produced using *cmdscale* on 1—proximity.

Accuracy, margins of classification, Cohen's Kappa, and area under the ROC (Receiver Operator Characteristic) curve (AUC) were used to evaluate the performance of classification models, as previously described (Enot et al. 2008). Models were deemed excellent overall if RF margins > 0.2 and AUC values, accuracy, and Cohen's Kappa > 0.8, as we have implemented previously (Enot et al. 2008; Lloyd et al. 2016).

Top ranked features contributing to the MDS models were extracted using re‐sampling methods and *p*‐values of False Positive rates (FPR ≤ 0.05).

### 2.9 Gas Chromatography–Mass Spectrometry (GC–MS)

Class pools of 50% methanol‐extracted urine were prepared, and 50 μL aliquots from each pool were dried directly into GC vials for derivatization. Pierce TriSil reagent was added to the sealed GC vials, which were then Whirlimixed. The samples underwent heating at 50°C for 20 min to facilitate trimethylsilylation (TMS), after which they were centrifuged and analyzed using a Perkin Elmer Clarus spectrometer equipped with a high‐polarity fused‐silica column (Perkin Elmer Elite—5MS, 30 m × 0.25 mm ID, 0.25 μm film thickness). Helium was used as the carrier gas at a flow rate of 1 mL/min. A 1 μL aliquot of each sample was injected via an injector maintained at 200°C. The TMS derivatives were separated using a temperature program starting at 160°C for 5 min, followed by a linear increase of 10°C/min to a final temperature of 300°C. Electron impact mass spectrometry of the column effluent was performed using a quadrupole ion filter system maintained at a constant 250°C throughout the analysis. The detector range was set from 100 to 650 amu, with a filament delay of 3 min.

### 2.10 NMR Analysis

A volume of 500 μL from the pooled urine samples was freeze‐dried and then reconstituted in deuterium oxide (D₂O). The prepared samples were analyzed using a 500 MHz Bruker Avance NMR spectrometer, equipped with a standard broadband observe probe. Data acquisition parameters included a spectral width of 10 ppm, with the chemical shifts referenced to the residual water peak in D₂O.

### 2.11 Plasma Extraction and Analysis of Short‐Chain Fatty Acids (SCFA), Detected by Gas Chromatography—Flame Ionization Detector (GC‐FID)

To prepare the internal standard solution, 50 mL of 20% orthophosphoric acid containing 20 mM 2‐ethylbutyric acid was pre‐chilled by placing it on ice. For plasma sample preparation, plasma stock samples previously stored at −80°C were slowly defrosted on ice. Once fully thawed, the samples were vortexed thoroughly to ensure homogeneity. A 200 μL aliquot of each sample was then transferred into labeled 1.5 mL Eppendorf tubes and kept on ice. To each sample, 50 μL of the pre‐chilled 20% orthophosphoric acid solution (containing 20 mM 2‐ethylbutyric acid) was added, and the mixture was vortexed.

Following the addition of the acid, the samples were shaken at 700 rpm, 4°C, for 20 min to allow for protein precipitation. After shaking, the samples were centrifuged at 13,000 rpm, 4°C, for 5 min. From the resulting supernatant, 150 μL was pipetted into a 200 μL GC micro vial. Each vial was then crimp capped and labeled on the metal cap.

Finally, the prepared samples were stored at 4°C until analysis on the same day. The analysis was conducted using a Thermo Scientific TRACE 1300 Series Gas Chromatograph equipped with a FID. The separation was performed on an HP‐FFAP column (15 m length × 0.53 mm I.D. × 1 μm film thickness) from Agilent, with a CP805305 Ultimate Plus deactivated fused silica guard column (5 m, 0.53 mm I.D.) also from Agilent. The injector temperature was set at 250°C, and 1 μL of each sample was injected into the system. Hydrogen was used as the carrier gas at a pressure of 5 psi, resulting in an approximate flow rate of 20 mL/min. The FID temperature was maintained at 250°C, with additional gases supplied as follows: make‐up gas (nitrogen) at 20 mL/min, hydrogen at 30 mL/min, and air at 300 mL/min. The system operated in split mode with a split flow of 40 mL/min.

The oven temperature program was as follows: an initial temperature of 75°C was held for 2 min, followed by a ramp of 5°C per minute up to 110°C. The temperature was then increased at 25°C per minute up to 220°C, where it was held for 3 min, resulting in a total run time of 19 min. For data analysis, SCFAs in samples were quantified using calibration curves.

Statistical analyses were conducted in R (version 4.3.0). To assess the effects of Kombucha consumption on plasma SCFA concentrations, we applied linear regression models for each SCFA (Acetic, Propionic, isoButyric, and isoValeric acids), including fixed effects for treatment group (Kombucha vs. Placebo), timepoint (Week 0 vs. Week 8), and their interaction (Treatment × Week). A significant interaction term (TreatmentKombucha × Week) was interpreted as evidence that the Kombucha group experienced a differential change in SCFA levels over time relative to Placebo. Statistical significance was set at *p* < 0.05, and marginal trends were noted at *p* < 0.1. Model diagnostics and coefficient estimates were used to evaluate the results.

## 3 Results

### 3.1 Recruitment

**FIGURE 1:**
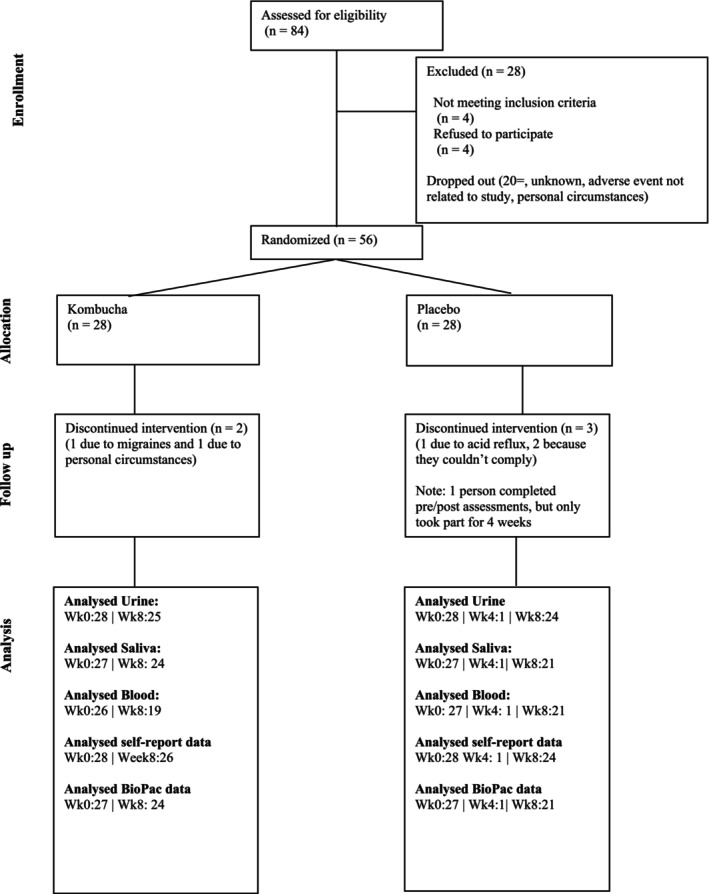
CONSORT diagram showing the flow of participants through each stage of the randomized trial.

Eighty‐four people provided information during the pre‐screening stage, with 61 progressing to the in‐person induction stage. After this point, 5 people decided they would not be able to commit to the trial, or they experienced a change in circumstances. As a result, 56 people in total enrolled in the trial (Figure 1).

The 56 individuals were randomized into either the Kombucha intervention group or the placebo group. At Week 0, there were 28 participants per group. The age range for the Kombucha group was 20–71, with the male to female ratio being: 7 males | 21 females. The age range for the Placebo group was 20–59, with the male to female ratio being: 9 males | 19 females. There were 5 dropouts between Week 0 and Week 8 (1 due to migraine, 1 due to acid reflux, and 3 due to a change in personal circumstances). At Week 8, the Kombucha group had 26 people (6 males | 20 females) and the Placebo group had 25 people (8 males | 18 females) (Table 1).

**TABLE 1:** Characteristics of the study participants at baseline.

Variable	Kombucha intervention		Placebo control	
	Mean	(SD)	Mean	(SD)
*Demographics*				
Total taking part in study [*n*]	28		28	
Gender Female [*n*]	21		19	
Gender Male [*n*]	7		9	
Age [years]	44	14.14	36.75	10.57
Age range (min–max) [years]	20–71		20–59	
*Anthropometrics*				
Weight [kg]	74.49	13.55	74.04	13.60
Height [cm]	1693.84	89.18	1686.46	85.46
BMI [kg m^−2^]	25.96	4.28	25.96	3.90
Waist circumference [cm]	85.18	12.43	87.66	10.18
Hip circumference [cm]	98.92	11.02	99.07	10.57
Waist to Hip ratio	0.86	0.10	0.89	0.11

*Note:* Data are presented as mean (SD) for continuous variables and as number of participants or percentage (%) for categorical variables. Body Mass Index (BMI) was calculated as [weight (kg)/height (m)^2^].

### 3.2 Urinary Analysis

**FIGURE 2:**
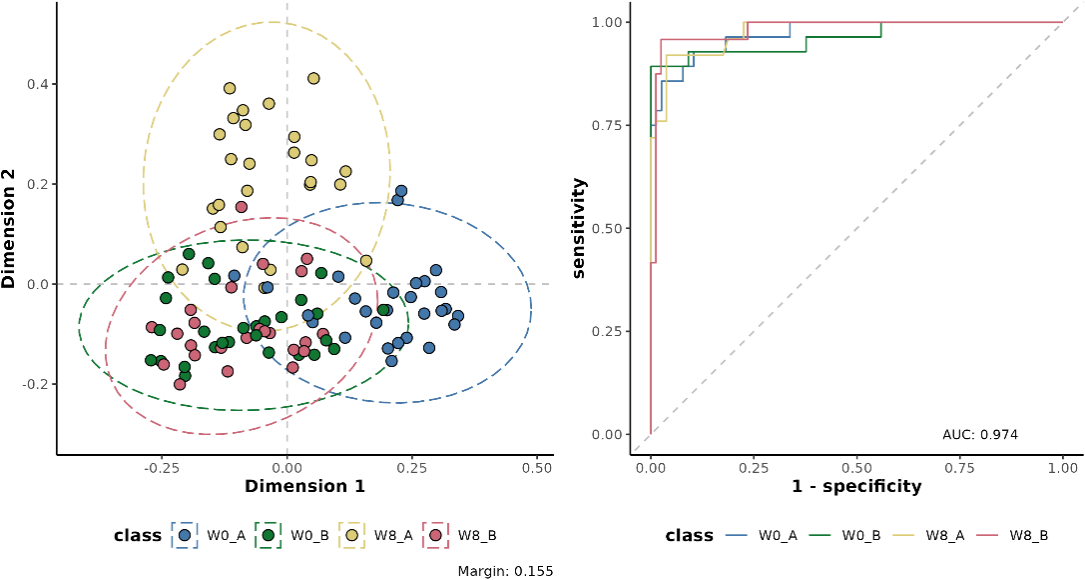
Multi‐dimensional scaling (MDS) of random forest (RF) proximity values of the flow infusion electrospray ionization mass spectrometry (FIE‐MS) fingerprint data. Where: Treatment: A = Kombucha, B = placebo, Week: 0 weeks = W0, 8 weeks = W8.

The processed FIE‐MS fingerprint data contained 5749 variables (134,581 features in the raw data). The processed data is in Appendix S2. RF classification models were plotted following MDS (Figure 2), showing separation of the week 8 intervention samples (Kombucha) compared to the baseline week 0 kombucha and placebo and the week 8 placebo samples (overlapping clusters observed).

The classification values for the baseline versus week 8 Kombucha showed, very clearly that the samples were chemically different: RF margin 0.25, AUC value 0.95, accuracy 0.87 and Cohen's kappa 0.74 (all above our defined values that we have implemented as ‘excellent’ previously (Enot et al. 2008; Lloyd et al. 2016); RF margins > 0.2 and AUC values and accuracy > 0.8 and Cohen's kappa > 0.6). The classification values for the other pairwise comparisons suggested that chemical changes were happening in the placebo arm too, however at a lesser extent than the kombucha arm. (Table 2).

**TABLE 2:** Classification values for the processed FIE‐MS fingerprint data.

Pair wise comparison	Accuracy	Cohen's kappa	RF margin	ROC AUC
W0_A ~ W0_B	0.83	0.65	0.23	0.91
W0_A ~ W8_A	0.87	0.74	0.25	0.95
W0_A ~ W8_B	0.84	0.67	0.23	0.92
W0_B ~ W8_A	0.89	0.78	0.20	0.95
W0_B ~ W8_B	0.89	0.78	0.15	0.96
W8_A ~ W8_B	0.87	0.74	0.21	0.94

*Note:* Where Accuracy, margins of classification, Cohen's Kappa and area under the ROC (Receiver Operator Characteristic) curve (AUC). (A is Kombucha and B is placebo.) = baseline and 8 = 8 weeks.

**FIGURE 3:**
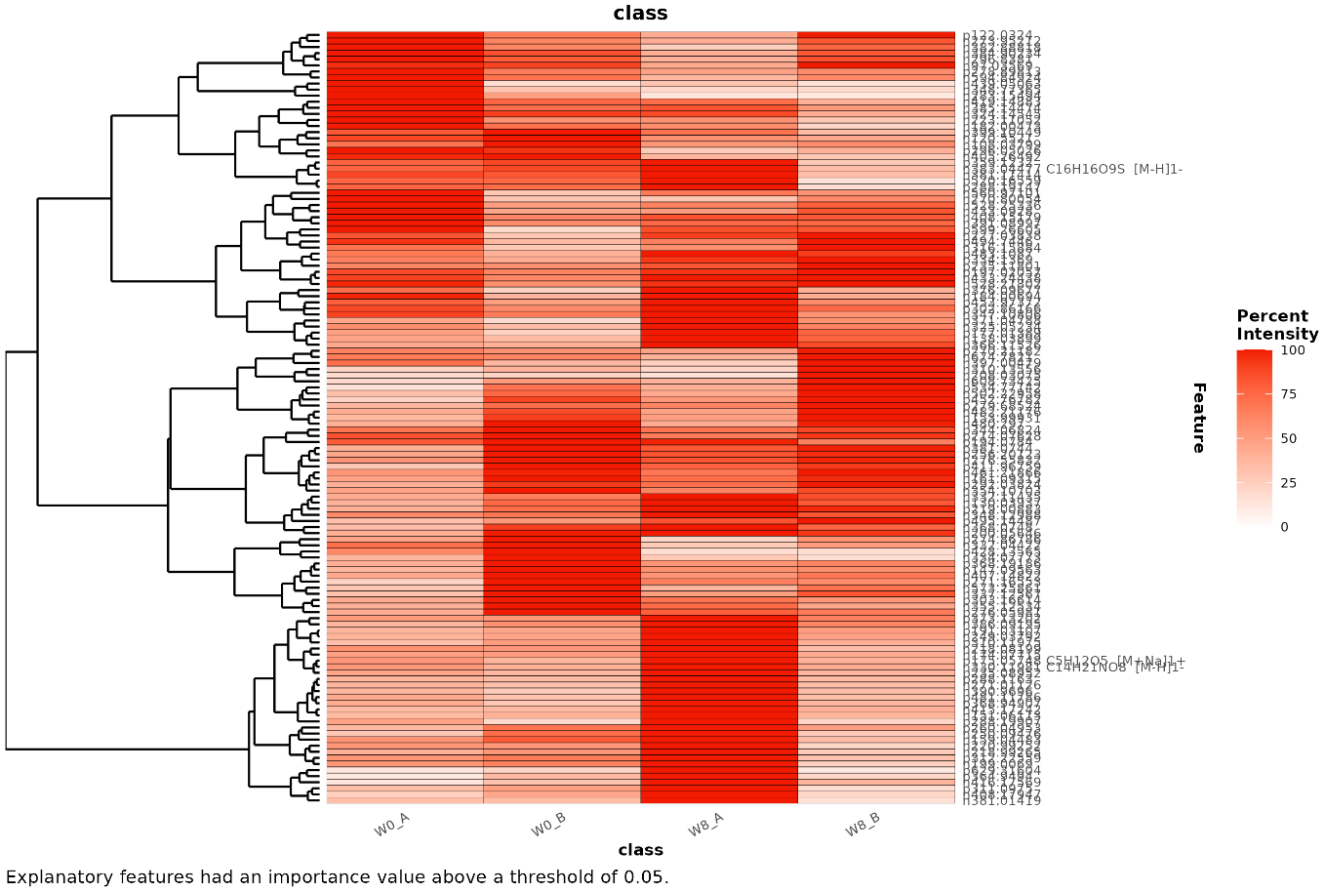
Heatmap of the top features and their relationships contributing to the discrimination. Where: Treatment: A = Kombucha, B = placebo, Week: 0 weeks = W0, 8 weeks = W8.

The top ranked features contributing to the MDS models were extracted using re‐sampling methods and *p*‐values of False Positive Rates (FPR ≤ 0.05). We used a heatmap to visualize the discriminatory features and the relationship between the features (Figure 3).

The top ranked features that showed elevation after kombucha consumption were plotted (Appendix S3) and queried using MZedDB, an interactive accurate mass annotation tool used to annotate signals by means of neutral loss and/or adduct formation rules (Draper et al. 2009). The putative molecular formulas, ionization products and annotations (level 2) are shown in Table 3.

### 3.3 Confirmation of Tentative Features

Arabitol, initially putatively identified as a pentitol elevated in post‐Kombucha urine samples, was confirmed through complementary analytical techniques. GC‐MS of the trimethylsilyl (TMS) derivatives showed that the retention time of the urinary pentitol peak (7.85 min) matched that of authentic Arabitol and was clearly distinguishable from xylitol (7.65 min) and ribitol (slightly longer retention time) under identical conditions (see Appendix S4). While the TMS derivatives of these pentitols yield nearly identical mass spectra, their ^1^H NMR spectra are distinct. To confirm the identity, 500 MHz proton NMR spectroscopy was performed. Despite the spectral complexity, characteristic resonances consistent with Arabitol were observed, including signals at 3.52 ppm (H1R, H1S, and H5S) and a possible H3 doublet at 3.42 ppm. These data confirmed the presence of Arabitol, supporting the GC–MS findings of its increased excretion following kombucha consumption.

Other signals from the top‐ranked features in Appendix S3, including those with unknown annotations, were not confirmed through structural analysis and were not pursued further in this study.

### 3.4 Plasma SCFA Analysis

**FIGURE 4:**
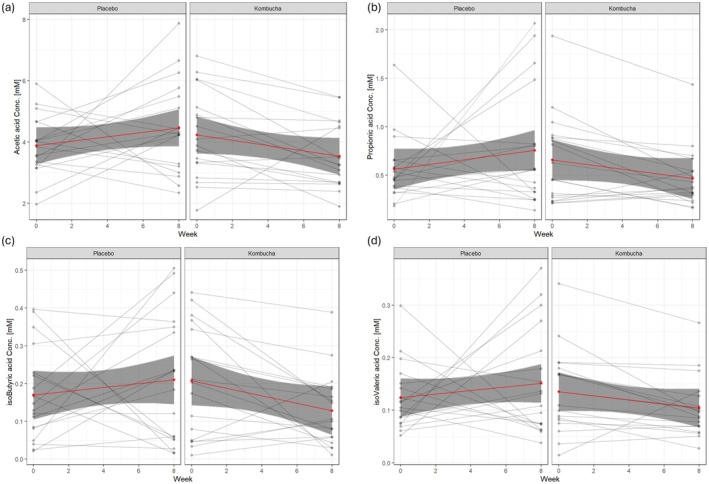
Plasma short‐chain fatty acid (SCFA) concentrations at baseline and post‐intervention. (a) Acetic acid, (b) Propionic acid, (c) isoButyric acid, and (d) isoValeric acid concentrations measured in plasma at Week 0 and Week 8. Lines represent individual participants, with panels representing treatment group (Placebo vs. Kombucha).

To complement urinary metabolomics and better understand microbial‐host interactions, we also examined plasma SCFAs. Quantification of plasma SCFAs was conducted using GC‐FID (full data in Appendix S5). A series of linear regression models with interaction terms (Treatment × Week) were used to assess changes over time between the Kombucha and Placebo groups. For each model, baseline values (intercepts) and time‐dependent changes (slopes) were compared across treatments (Appendix S6). For acetic acid, a significant interaction effect was observed (*β* = −0.1596, *p* = 0.037), with the Kombucha group showing a decline over time (Figure 4a). The estimated slope for Kombucha was −0.087 (0.0728–0.1596), suggesting a reduction in acetic acid levels after 8 weeks. With propionic acid, no significant interaction was detected (interaction *β* = −0.0040, *p* = 0.655), and slope differences between groups were negligible (Figure 4b). As for isoButyric acid, there was a marginal interaction effect (*β* = −0.0148, *p* = 0.069) indicating a small decrease in the Kombucha group. The slope for Kombucha was estimated at −0.0098 (Figure 4c). Lastly, for isoValeric acid, no significant changes were observed (interaction *β* = −0.0051, *p* = 0.381), with the Kombucha slope nearly flat (Figure 4d). These results suggest that Kombucha consumption may reduce plasma acetic and isoButyric acid levels, with acetic acid showing statistically significant effects and isoButyric acid showing a marginal trend.

## 4 Discussion

Global trends show that demand for functional food, including fermented food, is growing. A recent study showed that there is potential for further development of the functional food market as individuals are becoming more concerned about healthy eating and associate health benefits with functional food consumption (Remoundou et al. 2025). Kombucha, a fermented tea rich in bioactive compounds, continues to gather interest for its potential health benefits, yet its biochemical effects remain ununderstood. This study contributes to the growing body of evidence by exploring the urinary metabolome of individuals consuming kombucha daily for 8 weeks compared to a placebo drink. Using advanced metabolomics and machine learning techniques, we identified key metabolic shifts associated with kombucha intake, highlighting its capacity to modulate systemic metabolism and gut microbiota activity. In addition to urinary profiling, plasma analysis of SCFAs offered a targeted view of gut microbial activity. We observed a significant reduction in acetic acid and a marginal decrease in isoButyric acid in the Kombucha group, indicating a potential shift in microbial fermentation pathways. These findings align with the known production of SCFAs by gut microbes and suggest that kombucha may influence systemic metabolic outputs through microbiota modulation. These findings not only support other research (Fraiz et al. 2024; Wang et al. 2023) but also align with recent results by Ecklu‐Mensah et al. (2024), who reported microbiota shifts and enrichment of probiotic species following kombucha consumption, further underscoring its role as a functional beverage (Abbasi et al. 2024; Huang 2024).

We observed Arabitol to be elevated after kombucha consumption for 8 weeks. Arabitol, a sugar alcohol derived from arabinose or glucose, is naturally present in some fermented foods and can also arise as a metabolic byproduct of specific microbiological processes. Kombucha's fermentation, driven by diverse microbial strains, produces various metabolic products, including organic acids, ethanol, and potentially sugar alcohols such as arabitol (Wang et al. 2023). The detection of arabitol in urine could signify microbial activity related to kombucha consumption, as yeasts and lactic acid bacteria in kombucha are known to produce sugar alcohols under certain fermentation conditions. This aligns with the focus of this study on identifying microbial and metabolic interactions influenced by kombucha consumption.

We observed that methyluric acid, a derivative of uric acid, commonly associated with the metabolism of methylxanthines such as caffeine, a natural component of tea, elevated after kombucha consumption for 8 weeks. These metabolites, particularly 1‐methyluric acid, are regularly found in urine after consuming caffeine‐containing beverages such as tea and coffee (Martínez‐López et al. 2014). Their presence in urinary samples may also serve as a marker of trial compliance, for participants consuming kombucha.

Similar to the metabolomic shifts observed in the kombucha group in the Fraiz et al. (2024) study, our urinary metabolomics analysis revealed significant changes in features potentially linked to kombucha consumption, including polyphenol metabolites (Fraiz et al. 2024). One such metabolite is 3‐(4‐hydroxy‐3‐methoxyphenyl)propionic acid (HMPA), also known as dihydroferulic acid. HMPA is a hydroxycinnamic acid derivative resulting from the microbial transformation of dietary polyphenols like 4‐hydroxy‐3‐methoxycinnamic acid (ferulic acid) or naturally obtained from fermented foods (Tong et al. 2024). Studies have detected it in human plasma and urine following the consumption of polyphenol‐rich foods, such as berries and orange juice (Abe et al. 2023). HMPA may be a promising dietary supplement for muscle health and performance (Tong et al. 2024). Research has indicated that HMPA possesses health‐beneficial properties, including anti‐inflammatory and antioxidant effects. HMPA derived from dietary ferulic acid improved metabolic conditions in diet‐induced obese mice, suggesting potential health benefits (Ohue‐Kitano et al. 2019). The investigation of the pharmacokinetics of HMPA in rats found that orally administered HMPA was rapidly metabolized and widely distributed across various tissues, providing insight into the absorption and distribution of HMPA (Abe et al. 2023). A randomized controlled trial investigated the effects of whole‐grain wheat consumption in overweight and obese individuals with unhealthy dietary and lifestyle behaviors. The study found that participants who consumed whole‐grain wheat experienced a significant increase in serum dihydroferulic acid levels and a concomitant reduction in plasma tumor necrosis factor‐alpha (TNF‐α), an inflammatory marker. This suggests that the elevated dihydroferulic acid, resulting from whole‐grain wheat intake, may contribute to reduced inflammation and improved health outcomes (Vitaglione et al. 2015). Dihydroferulic acid regulates macrophage activation by inhibiting the release of pro‐inflammatory mediators and promoting anti‐inflammatory responses. The identification of HMPA in our study highlights the role of kombucha in enhancing polyphenol bioavailability and underscores its potential systemic health benefits.

Pyridoxine‐α‐glucoside is a glycosylated form of vitamin B6 commonly found in various plant‐based foods, including cereals and vegetables, and was another notable metabolite tentatively identified after kombucha consumption for 8 weeks. While tea leaves and kombucha contain B vitamins, including pyridoxine (vitamin B6), the specific glucoside form, pyridoxine‐α‐glucoside, has not been identified in tea or its fermented products (Teng et al. 2014; Kitwetcharoen et al. 2023). This finding may suggest a novel bioactive component associated with kombucha or its fermentation process, warranting further investigation.

Another feature tentatively identified (Table 3) was a sulphate conjugate of 5‐(3′,4′‐Dihydroxyphenyl)‐γ‐valerolactone (DHPV), which is a metabolite produced by the gut microbiota during the breakdown of dietary flavan‐3‐ols, compounds abundant in tea. After consuming flavan‐3‐ol‐rich foods or beverages, such as tea, these metabolites are absorbed into the bloodstream and afterwards excreted in urine (González‐Quilen et al. 2020; Urpi‐Sarda et al. 2009). The presence of DHPV metabolites suggests that kombucha consumption may influence gut microbiota activity and enhance the bioavailability of tea‐derived flavonoids, supporting its health‐promoting role.

**TABLE 3:** Putative molecular formulas and ionization products.

Feature	Ionization mode	*p*	Putative molecular formula and ionization product	Putative Identification
151.06113	Negative	0.0003	C_5_H_12_O_5_ [M‐H]^1−^	D‐Arabitol
218.99265	Negative	0.0071	C_6_H_6_N_4_O_3_ [M + K‐2H]^1−^	Methyluric acid
220.99252	Negative	0.0011	Unknown	Unknown
249.03792	Negative	0.0311	C_10_H_12_O_6_ [M + Na‐2H]^1−^	3‐(4‐hydroxy‐3‐methoxyphenyl)propionic acid
271.01126	Negative	0.0002	Unknown	Unknown
325.05234	Negative	0.0256	C_5_H_9_NO_3_S [2 M‐H]^1−^	Acetylcysteine
330.11981	Negative	0.0149	C_14_H_21_NO_8_ [M‐H]^1−^	Pyridoxine‐glucoside
390.96960	Negative	0.0484	Unknown	Unknown
415.17242	Negative	0.0369	C_21_H_30_O_7_ [M + Na‐2H]^1−^	Unknown
416.17569	Negative	0.0000	C_21_H_30_O_7_^13^C isotope [M + Na‐2H]^1−^	Unknown
175.05748	Positive	0.0251	C_5_H_12_O_5_ [M + Na]^1+^	D‐Arabitol
191.03107	Positive	0.0375	C_5_H_12_O_5_ [M + K]^1+^	D‐Arabitol
235.08952	Positive	0.0192	C_14_H_17_ClO_2_ [M + H‐H2O]^1+^	Unknown
288.11630	Positive	0.0024	Unknown	Unknown
311.09750	Positive	0.0191	C_13_H_18_N_2_O_4_ [M + 2Na‐H]^1+^	(Methoxybenzyl)glutamine
364.94940	Positive	0.0000	C_11_H_12_O_7_S [M + 2 K‐H]^1+^	(Dihydroxyphenyl)‐gamma‐valerolactone sulfate
481.11786	Positive	0.0000	C26H28O4 [M + 2 K‐H]^1+^	Unknown

Interestingly, methoxybenzyl glutamine, a metabolite detected in this study, has not been reported to be present in kombucha or other fermented foods before. Kombucha is rich in amino acids, including glutamine and its derivatives such as theanine, that are predominantly derived from tea leaves and the fermentation process (Jayabalan et al. 2010; Massoud et al. 2024); Sen et al. 2020).

These observations expand on findings from Fraiz et al. (2024) and align with emerging evidence from Ecklu‐Mensah et al. (2024), reinforcing kombucha's role in modulating gut‐derived metabolites. The reduction in plasma acetic acid and the marginal decrease in isoButyric acid that we observed support kombucha's influence on microbial fermentation, possibly through suppression of acetate‐ and branched‐chain SCFA‐producing pathways. Although propionic and isoValeric acids did not show significant changes, the directional trends align with the broader metabolomic shifts observed in both plasma and urine.

The observed biochemical changes may be mediated by kombucha‐derived polyphenols and microbial metabolites acting on the gut microbiota, subsequently modulating host metabolism. Fermented tea beverages are rich in bioactive compounds such as catechins, organic acids, and phenolic breakdown products, which can influence microbial fermentation pathways and systemic metabolic responses (Tong et al. 2024; Ohue‐Kitano et al. 2019). The reduction in SCFAs like acetic acid may reflect shifts in microbial populations or fermentation efficiency, possibly due to polyphenol–microbiome interactions.

Despite the promising findings, this study has several limitations. First, while urinary metabolomics provides a non‐invasive and rich dataset for evaluating metabolic responses, it reflects only a subset of systemic biochemical changes. The reliance on tentative metabolite annotations without validation using reference standards introduces uncertainty into some compound identifications. The study's scope was limited to urinary profiling fingerprinting and plasma SCFA analysis; possible integration with serum metabolomics and fecal sample microbiome sequencing would offer a more comprehensive picture of kombucha's physiological impact. Additionally, while the randomized controlled design strengthens the findings, the cohort was female dominated, and the study duration and sample size may have also limited the detection of more subtle or longer‐term effects. Future studies should aim to validate these findings using larger cohorts, extended intervention periods, and multi‐omics integration. It is important to note that this study did not test the microbial viability or strain composition of the kombucha used. Therefore, no conclusions can be drawn about its probiotic properties, and generalization to other kombucha products is limited. Participants were not required to restrict their usual caffeine intake (e.g., tea/coffee). This could have introduced some inter‐individual variation in methyluric acid excretion, which we acknowledge as a limitation. Future studies may consider dietary standardization or detailed caffeine intake recording.

## 5 Conclusion

The integration of untargeted urinary metabolomics with machine learning, particularly RF classification, enabled the identification of distinct chemical signatures between the kombucha and placebo groups. These findings offer novel insights into the potential systemic effects of kombucha consumption, suggesting it may influence host metabolism and gut microbial activity. The inclusion of plasma SCFA analysis further supports kombucha's capacity to modulate gut‐derived metabolic outputs.

This study highlights the strength of combining advanced computational techniques with high‐throughput biochemical profiling to detect subtle yet meaningful metabolic changes. However, as several metabolite annotations remain putative, these findings should be interpreted as exploratory. Future validation with authentic standards and integrated microbiome and serum metabolomic analyses is warranted.

Building on the emerging literature—including studies by Fraiz et al. (2024) and Ecklu‐Mensah et al. (2024)—this work contributes to the growing evidence that fermented foods may modulate systemic biochemistry. While preliminary, the results provide a valuable foundation for future research into traditional kombucha's functional role in diet and health.

## Author Contributions

**Amanda J. Lloyd:** conceptualization (equal), formal analysis (equal), funding acquisition (equal), investigation (equal), methodology (equal), project administration (lead), supervision (equal), validation (equal), writing – original draft (lead), writing – review and editing (equal). **Robert J. Nash:** formal analysis (equal), validation (equal), writing – original draft (supporting), writing – review and editing (equal). **Alexander N. W. Taylor:** conceptualization (equal), formal analysis (equal), funding acquisition (supporting), investigation (supporting), methodology (supporting), writing – original draft (supporting), writing – review and editing (supporting). **Alina Warren‐Walker:** conceptualization (equal), formal analysis (equal), investigation (equal), methodology (equal), validation (equal), writing – review and editing (supporting). **Courtney Davies:** conceptualization (supporting), formal analysis (equal), investigation (equal), methodology (equal), validation (equal). **M. J. Pilar Martinez Martin:** formal analysis (supporting), investigation (supporting), methodology (supporting), validation (supporting). **Odin M. Moron‐Garcia:** validation (equal). **Alison Watson:** methodology (equal), validation (supporting). **Laura Lyons:** methodology (equal), validation (equal). **Mark J. Pavey:** funding acquisition (supporting), writing – review and editing (supporting). **Thomas Wilson:** formal analysis (supporting), investigation (supporting). **Manfred Beckmann:** formal analysis (supporting), investigation (supporting), methodology (supporting), supervision (supporting), validation (supporting), writing – review and editing (supporting). **Kyriaki Remoundou:** funding acquisition (equal), investigation (supporting), writing – original draft (supporting), writing – review and editing (supporting). **Nigel Holt:** funding acquisition (equal), supervision (equal), writing – original draft (equal), writing – review and editing (equal).

## Ethics Statement

Ethical approval was gained on the 07/02/2024 by the Research Ethics Group of Aberystwyth University, reference number: 25618. The trial is registered in the ISRCTN registry (https://www.isrctn.com/) under registry number ISRCTN70525386. The Declaration of Helsinki and the Recommendations for the Conduct, Reporting, Editing and Publication of Scholarly Work in Medical journals guidelines have been followed.

## Conflicts of Interest

M.J. Pavey, listed as an author, is the owner and producer at Conwy Kombucha Ltd., which supplied the kombucha used in the study. Robert J. Nash is affiliated with Phytoquest Ltd., one of the industry partners in the project. The project was led by Aberystwyth University and funded by Innovate UK Better Food for All (10074259). Conwy Kombucha Ltd. and Phytoquest Ltd. were industry collaborators but had no role in the study design, data collection, statistical analysis, interpretation of data, or decision to submit the manuscript for publication. The authors declare that there are no other competing financial interests.

## Supporting information


**Appendix S1:** Ingredient composition of the trial kombucha beverage and the placebo flavored water.

**Appendix S2:** Processed urinary metabolomic fingerprint data acquired by Flow Infusion Electrospray Ionization Mass Spectrometry (FIE‐MS).

**Appendix S3:** Top‐ranked urinary metabolite features that increased following kombucha consumption, plotted after Random Forest feature selection.

**Appendix S4:** Confirmation of urinary arabitol signals using Gas Chromatography (GC) and 500 MHz Proton Nuclear Magnetic Resonance (1H NMR) spectroscopy.

**Appendix S5:** Quantification of plasma short‐chain fatty acids (SCFAs) measured by Gas Chromatography–Flame Ionization Detection (GC‐FID).

**Appendix S6:** Linear regression models assessing the effects of kombucha consumption on plasma short‐chain fatty acids (SCFAs).

## Data Availability

The urine metabolomics data (processed positive and negative mode) and plasma SCFA data are available in Appendices S4 and S5, respectively.
