# Supplementary material for: Daily Consumption of Kombucha Influences the Urinary and Plasma Metabolome in a Healthy Human Cohort
Source: Food Sci Nutr. 2025 Oct 13;13(10):e71020. doi: 10.1002/fsn3.71020 (PMC12516354; doi:10.1002/fsn3.71020)
Supplement: Supplementary file 1 — Appendix S1: Ingredient composition of the trial kombucha beverage and the placebo flavored water. [file FSN3-13-e71020-s003.docx]

**Appendix 1**

**Kombucha**
Water, Live Kombucha Culture*, Cane Sugar*, Green Tea*, Black Tea*.  *organic

**Placebo**
Water, Cherry Syrup 2% [Sour Cherry Juice Concentrate (2.29%), Red Grape Juice (2.23%), Preservative: (Malic Acid), Natural Colour: (Carantho), Natural Flavouring: (Red Grape, Sour Cherry), Preservative: (Potassium Sorbate), Antioxidant (Ascorbic Acid (Vitamin C)), Hibiscus]

The Kombucha contains green tea, which contains low amounts of caffeine. The products are not hazardous. The products are free of the following components and their products thereof: cereals containing gluten, crustaceans, eggs, fish, peanuts, soybeans, milk (including lactose), nuts, celery, mustard, sesame seeds, sulphur dioxide and sulphite, lupin and molluscs, in compliance with Regulation (EC) No. 1169/2011. The products do not contain added artificial colorants or E numbers. The kombucha and placebo are vegan, and they are produced and stored in a food-grade facility.
