# Supplementary figures and images for: Daily Consumption of Kombucha Influences the Urinary and Plasma Metabolome in a Healthy Human Cohort
Source: Food Sci Nutr. 2025 Oct 13;13(10):e71020. doi: 10.1002/fsn3.71020 (PMC12516354; doi:10.1002/fsn3.71020)

## Slide 1
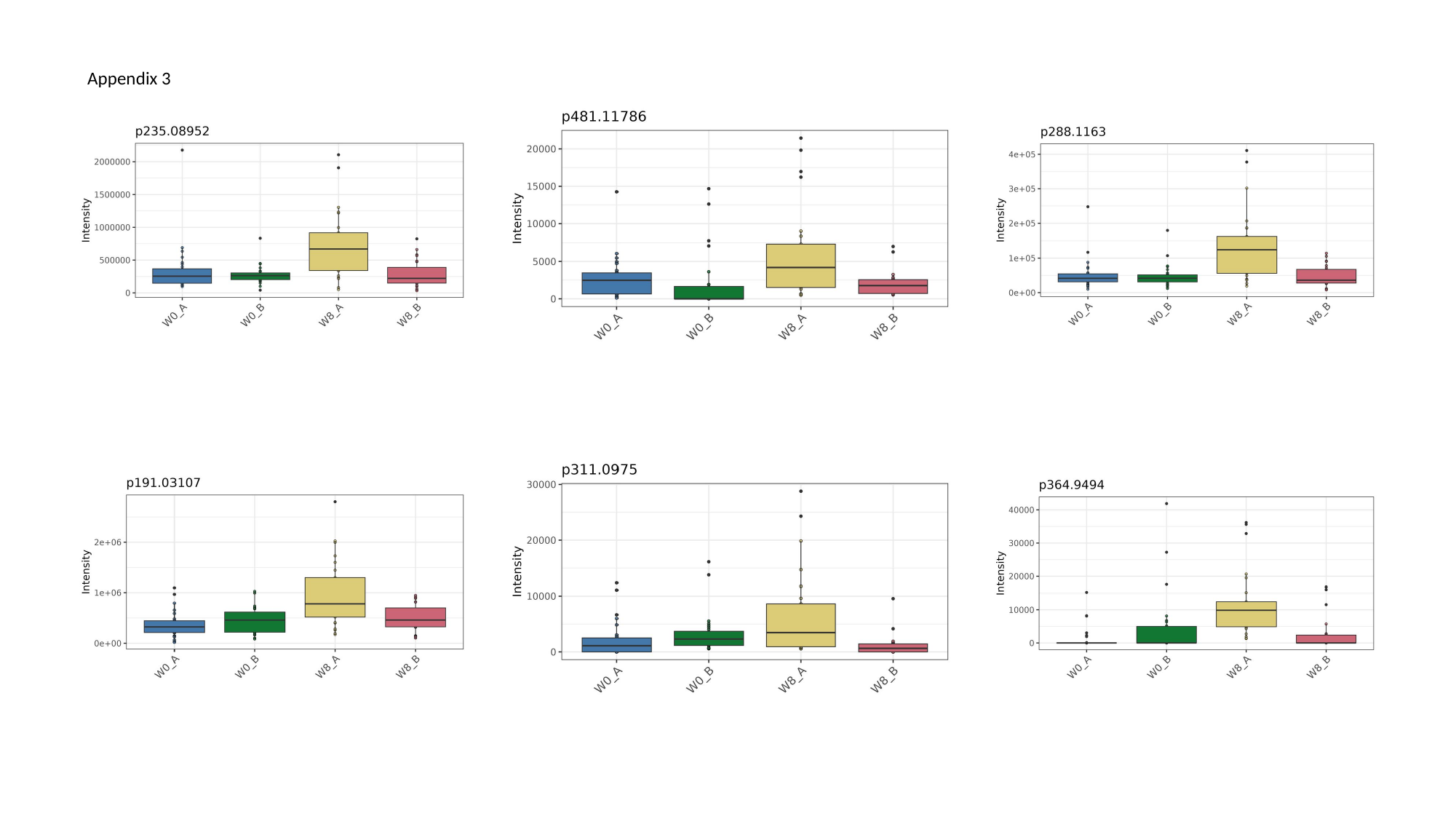

Appendix 3

## Slide 2
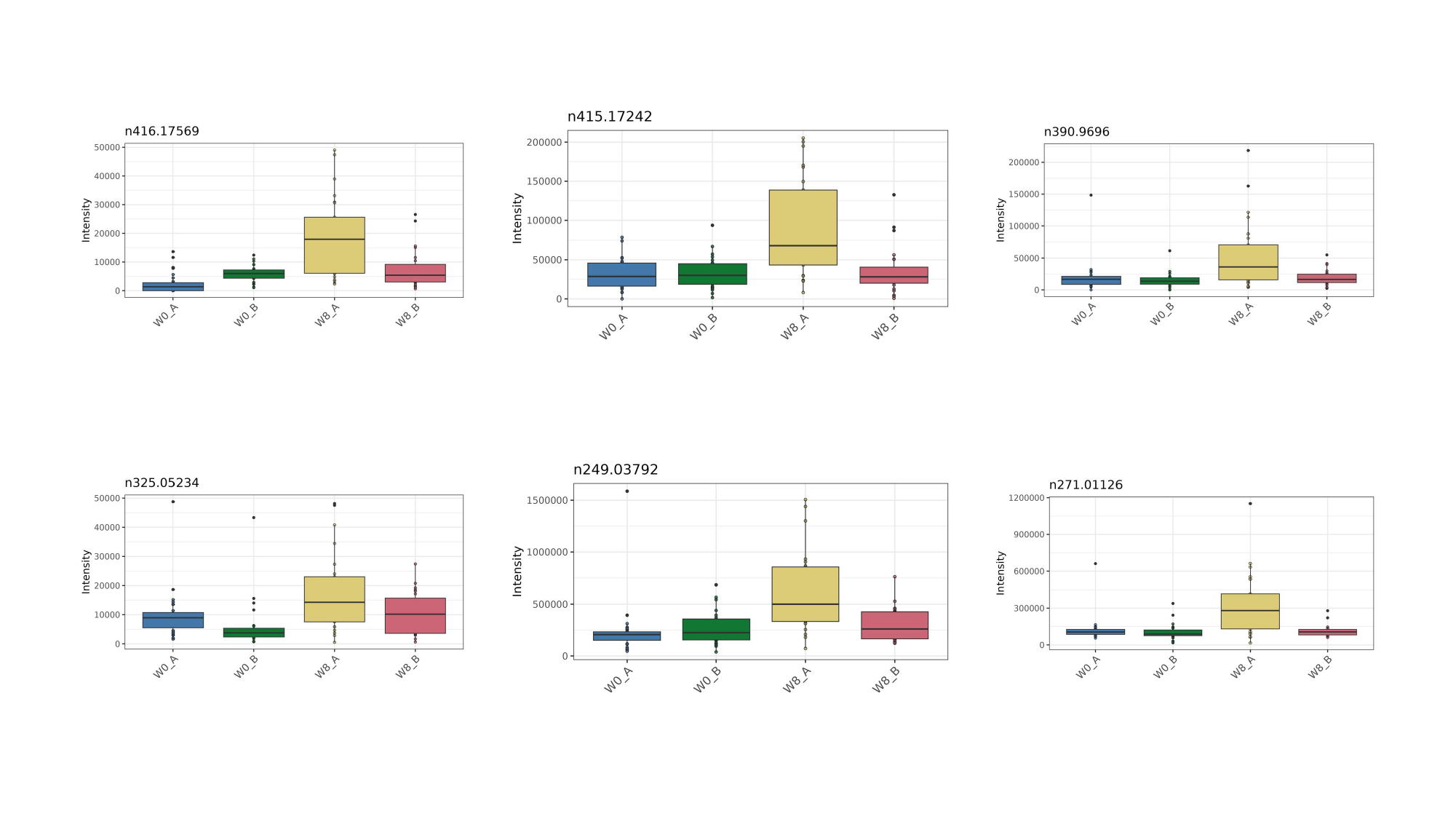

## Slide 3
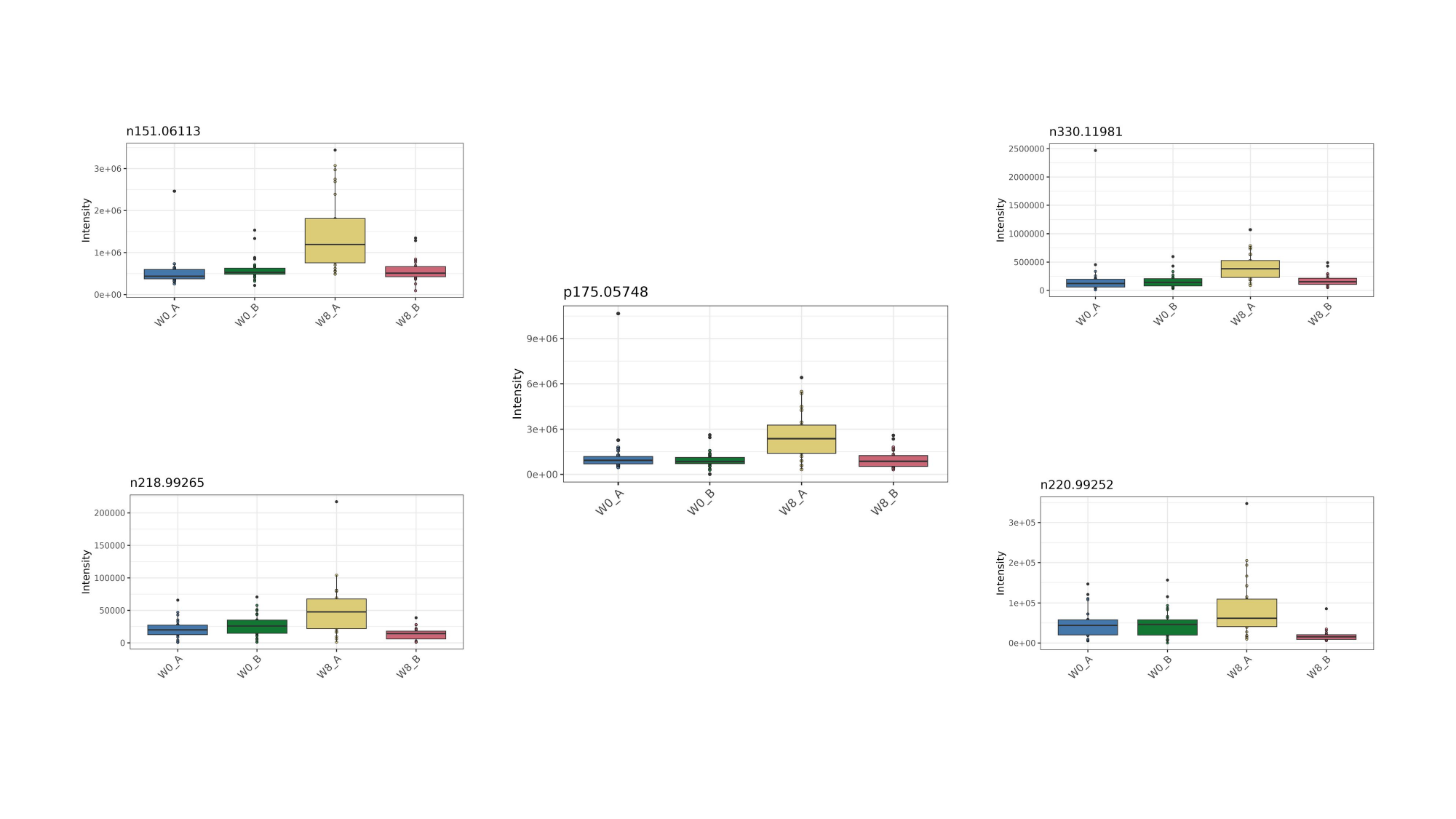

Supplement: Supplementary file 3 — Appendix S3: Top‐ranked urinary metabolite features that increased following kombucha consumption, plotted after Random Forest feature selection. [file FSN3-13-e71020-s002.pptx]
