# Supplementary material for: Daily Consumption of Kombucha Influences the Urinary and Plasma Metabolome in a Healthy Human Cohort
Source: Food Sci Nutr. 2025 Oct 13;13(10):e71020. doi: 10.1002/fsn3.71020 (PMC12516354; doi:10.1002/fsn3.71020)
Supplement: Supplementary file 4 — Appendix S4: Confirmation of urinary arabitol signals using Gas Chromatography (GC) and 500 MHz Proton Nuclear Magnetic Resonance (1H NMR) spectroscopy. [file FSN3-13-e71020-s001.docx]

**Appendix 4**

**GCMS chromatogram showing xylitol (7.65 minutes) and arabitol (7.85 minutes)**

**Mass spectra (tms) of xylitol and arabitol**

**500 MHz proton NMR spectrum of pooled pre-Kombucha urine**

Mannitol signals arabitol signals

**
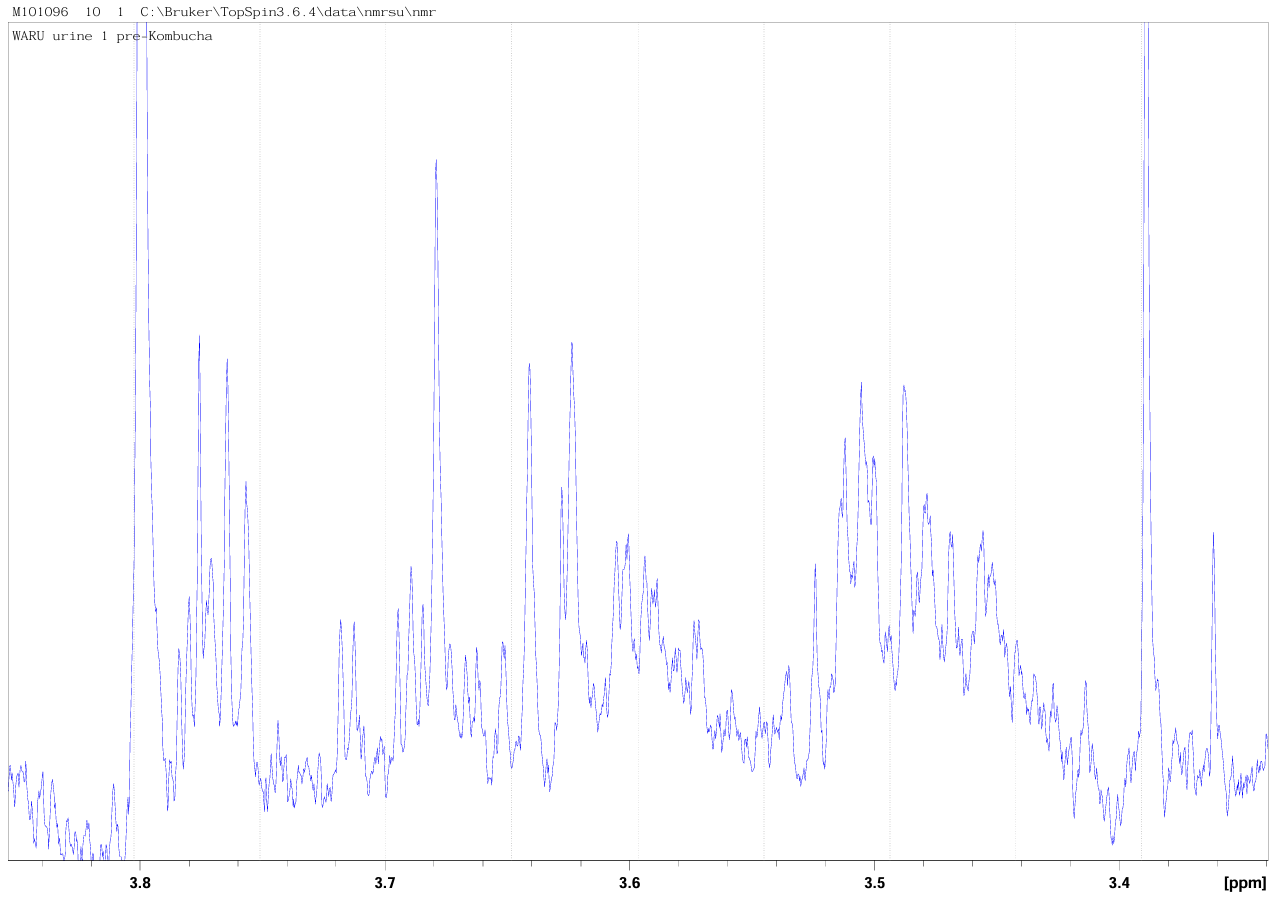
**
