# Supplementary material for: Daily Consumption of Kombucha Influences the Urinary and Plasma Metabolome in a Healthy Human Cohort
Source: Food Sci Nutr. 2025 Oct 13;13(10):e71020. doi: 10.1002/fsn3.71020 (PMC12516354; doi:10.1002/fsn3.71020)
Supplement: Supplementary file 6 — Appendix S6: Linear regression models assessing the effects of kombucha consumption on plasma short‐chain fatty acids (SCFAs). [file FSN3-13-e71020-s004.docx]

**Appendix 6: Linear Model Analysis of Plasma SCFAs**

Linear regression models were used to assess changes in plasma short-chain fatty acids (SCFAs) over time between Kombucha and Placebo groups. Each model included fixed effects for treatment group, time (Week 0 vs. Week 8), and their interaction (Treatment * Week).

**A. Acetic Acid**

| **Coefficient** | **Estimate** | **Std. Error** | ***t*-value** | ***p*-value** |
| --- | --- | --- | --- | --- |
| (Intercept) | 3.883 | 0.300 | 12.939 | < 2e-16 *** |
| Treatment (Kombucha) | 0.346 | 0.424 | 0.816 | 0.417 |
| Week | 0.073 | 0.053 | 1.372 | 0.174 |
| Treatment × Week | -0.160 | 0.075 | -2.128 | **0.037** * |

## Residual standard error: 1.273 on 68 degrees of freedom

## Multiple R-squared: 0.07497, Adjusted R-squared: 0.03416

## F-statistic: 1.837 on 3 and 68 DF, p-value: 0.1487

## For Placebo, the slope is 0.0728055555555558

## For Kombucha, the slope is -0.0868333333333334

**Interpretation**: Acetic acid showed a significant interaction effect. Kombucha participants exhibited a reduction over 8 weeks (*p* = 0.037), with a net slope of -0.087.

**B. Propionic Acid**

| **Coefficient** | **Estimate** | **Std. Error** | ***t*-value** | ***p*-value** |
| --- | --- | --- | --- | --- |
| (Intercept) | 0.926 | 0.058 | 15.857 | < 2e-16 *** |
| Treatment (Kombucha) | 0.025 | 0.081 | 0.311 | 0.757 |
| Week | -0.002 | 0.007 | -0.366 | 0.716 |
| Treatment × Week | -0.004 | 0.009 | -0.449 | 0.655 |

## Residual standard error: 0.4448 on 68 degrees of freedom

## Multiple R-squared: 0.05853, Adjusted R-squared: 0.017

## F-statistic: 1.409 on 3 and 68 DF, p-value: 0.2476

For Placebo, the slope is 0.0239583333333334

For Kombucha, the slope is -0.0240138888888889

**Interpretation**: No significant treatment or interaction effects were observed for propionic acid.

**C. isoButyric Acid**

| **Coefficient** | **Estimate** | **Std. Error** | ***t*-value** | ***p*-value** |
| --- | --- | --- | --- | --- |
| (Intercept) | 0.142 | 0.018 | 7.824 | < 2e-10 *** |
| Treatment (Kombucha) | 0.016 | 0.025 | 0.647 | 0.520 |
| Week | 0.005 | 0.006 | 0.885 | 0.379 |
| Treatment × Week | -0.015 | 0.008 | -1.847 | 0.069 |

## Residual standard error: 0.1364 on 68 degrees of freedom

## Multiple R-squared: 0.05876, Adjusted R-squared: 0.01723

## F-statistic: 1.415 on 3 and 68 DF, p-value: 0.2459

For Placebo, the slope is 0.0050277777777778

For Kombucha, the slope is -0.0098125

**Interpretation**: isoButyric acid showed a marginal interaction effect (*p* = 0.069), with the Kombucha group experiencing a slight decline.

**D. isoValeric Acid**

| **Coefficient** | **Estimate** | **Std. Error** | ***t*-value** | ***p*-value** |
| --- | --- | --- | --- | --- |
| (Intercept) | 0.258 | 0.022 | 11.722 | < 2e-16 *** |
| Treatment (Kombucha) | 0.002 | 0.031 | 0.064 | 0.949 |
| Week | 0.010 | 0.007 | 1.456 | 0.151 |
| Treatment × Week | -0.005 | 0.006 | -0.879 | 0.381 |

## Residual standard error: 0.07769 on 68 degrees of freedom

## Multiple R-squared: 0.04859, Adjusted R-squared: 0.006613

## F-statistic: 1.158 on 3 and 68 DF, p-value: 0.3324

For Placebo, the slope is 0.00336805555555556

For Kombucha, the slope is -0.00385416666666667

**Interpretation**: No significant treatment or interaction effects were detected for isoValeric acid.
